# Interaction of Bacterial Phenazines with Colistimethate in Bronchial Epithelial Cells

**DOI:** 10.1128/AAC.02349-17

**Published:** 2018-07-27

**Authors:** Valeri V. Mossine, Deborah L. Chance, James K. Waters, Thomas P. Mawhinney

**Affiliations:** a Department of Biochemistry, University of Missouri, Columbia, Missouri, USA; b Experiment Station Chemical Laboratories, University of Missouri, Columbia, Missouri, USA; c Department of Molecular Microbiology and Immunology, University of Missouri, Columbia, Missouri, USA; d Department of Child Health, University of Missouri, Columbia, Missouri, USA

**Keywords:** polymyxins, colistimethate sodium, pyocyanin, cytotoxicity, synergism, phenazines

## Abstract

Multidrug-resistant bacterial infections are being increasingly treated in clinics with polymyxins, a class of antibiotics associated with adverse effects on the kidney, nervous system, or airways of a significant proportion of human and animal patients. Although many of the resistant pathogens display enhanced virulence, the hazard of cytotoxic interactions between polymyxin antibiotics and bacterial virulence factors (VFs) has not been assessed, to date. We report here the testing of paired combinations of four Pseudomonas aeruginosa VF phenazine toxins, pyocyanin (PYO), 1-hydroxyphenazine (1-HP), phenazine-1-carboxylic acid (PCA), and phenazine-1-carboxamide (PCN), and two commonly prescribed polymyxin drugs, colistin-colistimethate sodium (CMS) and polymyxin B, in three human airway cell lines, BEAS-2B, HBE-1, and CFT-1. Cytotoxicities of individual antibiotics, individual toxins, and their combinations were evaluated by the simultaneous measurement of mitochondrial metabolic, total transcriptional/translational, and Nrf2 stress response regulator activities in treated cells. Two phenazines, PYO and 1-HP, were cytotoxic at clinically relevant concentrations (100 to 150 μM) and prompted a significant increase in oxidative stress-induced transcriptional activity in surviving cells. The polymyxin antibiotics arrested cell proliferation at clinically achievable (<1 mM) concentrations as well, with CMS displaying surprisingly high cytotoxicity (50% effective dose [ED_50_] = 180 μM) in BEAS-2B cells. The dose-response curves were probed by a median-effect analysis, which established a synergistically enhanced cytotoxicity of the PYO-CMS combination in all three airway cell lines; a particularly strong effect on BEAS-2B cells was observed, with a combination index (CI) of 0.27 at the ED_50_. PCA, PCN, and 1-HP potentiated CMS cytotoxicity to a smaller extent. The cytotoxicity of CMS could be reduced with 10 mM *N*-acetyl-cysteine. Iron chelators, while ineffective against the polymyxins, could rescue all three bronchial epithelial cell lines treated with lethal PYO or CMS-PYO doses. These findings suggest that further evaluations of CMS safety are needed, along with a search for means to moderate potentially cytotoxic interactions.

## INTRODUCTION

The emergence of multidrug-resistant (MDR) bacterial pathogens in the last decades has presented significant challenges to clinicians, limiting the choices of effective antimicrobial agents available to treat MDR infections (1). Polymyxin B (PMB) and polymyxin E (colistin [CST]) are well-known and effective antibiotics against Gram-negative bacteria. They were developed over 60 years ago but were, until recently, largely abandoned, except for inhalations and topical use, due to nephrotoxic and neurotoxic side effects (2). Recently, these polymyxins have been reemployed to help with the clinical treatment of MDR infections, and significant effort was devoted to closing knowledge gaps in the understanding of their pharmacokinetics, pharmacodynamics, or mechanisms of toxicity (3–5). Major clinical targets for polymyxins include chronic Gram-negative bacterial, e.g., Pseudomonas aeruginosa, infections in airways of patients with cystic fibrosis (CF) and, increasingly, ventilator-associated MDR pneumonias (6, 7). The pharmaceutical polymyxin E form in these treatments is colistimethate sodium (CMS), a prodrug of CST, with delivery either parenterally or by aerosol inhalation (7). For years, CMS has been regarded as the safer form of polymyxin drugs (2, 5, 8). However, there are a number of reports citing detrimental side effects of inhaled CMS in airways (9, 10), including lethality from acute respiratory distress syndrome (11). For example, in two randomized clinical trials (12, 13), inhalation of CMS in the forms of a nebulized solution and powder caused repeated treatment-related adverse events in the respiratory system of 35% and 82% of CF patients, respectively. It is not currently known by which mechanism(s) inhaled CMS may produce pulmonary side effects. So far, the only available relevant *in vitro* study (8) suggested that formed colistin (14), rather than its parent CMS, may trigger apoptosis in alveolar epithelial cells via extrinsic death receptor and intrinsic mitochondrial pathways.

Clinical isolates of P. aeruginosa, characterized from acute and chronic pulmonary infections, release a number of specialized multifunctional metabolites. Pyocyanin (PYO) is one of the most common and is familiar as blue pigment in sputa (15, 16). PYO is recognized as a virulence factor (VF), because this molecule is helpful to P. aeruginosa colonization in several ways, including cytotoxicity toward host ciliated epithelial cells (17), immune cells (18), and competing microbes (19). The chemical structure of PYO allows for ready intracellular entry, where it acts as a redox catalyst, induces oxidative stress, and depletes intracellular NAD(P)H and glutathione (20, 21). In addition to PYO, P. aeruginosa also releases a number of phenazine molecules that are structurally and functionally related to PYO (19). The cytotoxicity of PYO and other phenazines to airway epithelial cells (15, 17, 22, 23), their ability to cause bronchoconstriction in animals (24), and the negative correlation between sputum phenazine concentrations and pulmonary function in CF (16) have been established.

For the ongoing practice of polymyxin-based therapies, the identification of potentially toxic drug interactions is an important safety issue. Given that in patients with P. aeruginosa lung infections, airway epithelial cells are in a position to encounter relatively high concentrations of both pseudomonal phenazines (15) and the inhaled antibiotics CMS and formed colistin (14), we asked whether such an encounter poses a potential hazard. Our study thus aimed to evaluate cytotoxic interactions between polymyxins and bacterial phenazines, using a recently developed cellular stress response reporter platform (25). We present here our initial data on the cytotoxicity of colistin, CMS, polymyxin B, four common P. aeruginosa phenazines, and combinations of the phenazines and polymyxins in three human bronchial epithelial cell lines. We demonstrate that CMS can interact with PYO by way of producing a synergistically enhanced cytotoxic effect against airway epithelial cells *in vitro* and identify inhibitors capable of modulating this cytotoxicity.

## RESULTS

We first determined the individual cytotoxicities of polymyxin drugs and pseudomonal phenazines using assays based on two different cellular activities: (i) mitochondrial reduction of resazurin (26) and (ii) continuous production of destabilized green fluorescent protein (GFP) under the control of the constitutive elongation factor 1α (EF-1α) promoter (25) in the reporter cell line BEAS-2B.R05Z. These assays do not distinguish between live and dead cells, as they measure averaged mitochondrial metabolic and transcriptional/translational activities in the wells, respectively. Since all treatment experiments were conducted under conditions of cellular confluence in the wells, the data obtained in these assays are expressed in terms of cellular functional responses rather than cell numbers. For cells harboring the stably transfected reporter construct, which responds to the transcriptional activity of the cellular stress response master regulator Nrf2 (25), this approach allowed the simultaneous determination of mitochondrial metabolic, transcriptional/translational, and Nrf2 antioxidant/recovery activities in toxin-stressed cells from the very same wells. The cytotoxicity profiling experiments with two additional bronchial epithelial cell lines, HBE-1 and CFT-1, were limited to the resazurin assay only.

### Effects of P. aeruginosa phenazines on viability and Nrf2 activity in bronchial epithelial cells.

**FIG 1:**
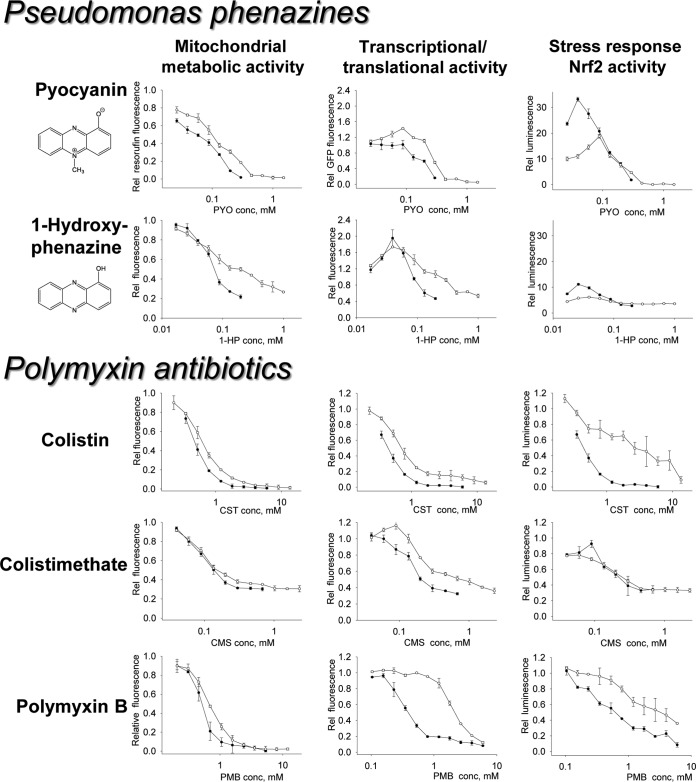
Cytotoxicity of polymyxins and P. aeruginosa phenazines in BEAS-2B cells. The mitochondrial metabolic activity was evaluated fluorimetrically by measuring rates of resazurin reduction. Transcriptional/translational activity was assessed by the determination of destabilized GFP in cell lysates. The relative activity of the stress-responsive transcriptional factor Nrf2 was calculated by normalizing the luminescence readings with the GFP fluorescence values from the same wells. The open and closed circles correspond to 12-h (plus 12-h recovery) and 24-h drug exposure schedules. The error bars are standard deviations (SDs) for at least 3 biological samples. Conversion formulae for the drug concentration are as follows: 1 μM equals 1.4 mg/liter colistin sulfate, 1.45 mg/liter polymyxin B sulfate, or 1.75 mg/liter colistimethate sodium.

The calculated values of the phenazine concentrations producing a 50% inhibitory effect in the BEAS-2B, HBE-1, and CFT-1 lines are given in Table 1. Of the four common P. aeruginosa phenazines pyocyanin, 1-hydroxyphenazine (1-HP), phenazine-1-carboxylic acid (PCA), and phenazine-1-carboxamide (PCN), only PYO and 1-HP displayed significant cytotoxicities toward bronchial epithelial cells within physiologically achievable concentrations (such as up to 130 μM PYO being observed in sputa of patients with CF and bronchiectasis [15]). There was a large, up to 35-fold, increase in Nrf2 transcription factor activity in BEAS-2B cells treated with PYO (Fig. 1), which achieved its maximum at subtoxic (<100 μM) concentrations of the phenazine. A similar, albeit less intense, Nrf2 activation profile was observed for cells treated with 1-HP. PCA and PCN caused only minor decreases in cellular viability after 24 h of exposure, and PCA induced a small increase in the Nrf2 activity at the 240 μM dose range (see Fig. S1 in the supplemental material). Since dose-response curves produced by 24-h exposures of PCA and PCN to cells showed small effects at 240 μM and due to limited solubility of these phenazines, no further attempts to determine their cytotoxicities were undertaken.

**TABLE 1:** Median-effect dose parameter ED_50_ for phenazines and polymyxins in human bronchial epithelial cell lines^*a*^

Treatment	Mean ED_50_ (μM) ± SD for cell line (assay)					
	BEAS-2B (resazurin reduction)		BEAS-2B (GFP production)		HBE-1 (resazurin reduction) for 24 h	CFT-1 (resazurin reduction) for 24 h
	24 h	12 h	24 h	12 h		
Phenazines						
PYO	87 ± 3	115 ± 9	188 ± 5	347 ± 30	164 ± 27	150 ± 17
1-HP	82 ± 3	212 ± 12	174 ± 5	937 ± 113	168 ± 6	176 ± 6
PCA	>240	NA	>240	NA	>240	>240
PCN	>240	NA	>240	NA	>240	>240
Polymyxins						
Colistimethate	180 ± 11	202 ± 10	268 ± 16	776 ± 36	252 ± 26	333 ± 14
Colistin	454 ± 21	710 ± 52	446 ± 26	778 ± 28	141 ± 3	91 ± 2
Polymyxin B	526 ± 43	686 ± 38	539 ± 51	2,419 ± 88	111 ± 7	103 ± 4

a Conversion formulae for the drug concentrations are as follows: 1 μM equals 1.4 mg/liter colistin sulfate, 1.45 mg/liter polymyxin B sulfate, or 1.75 mg/liter colistimethate sodium. NA, not applicable.

As shown in Fig. 1, the dose-response curves for BEAS-2B cells treated with PYO and 1-HP were recorded for both 24-h exposure and 12-h exposure/12-h recovery schedules. While the mitochondrial metabolic (resazurin reduction) activity in cells treated with PYO or 1-HP decreased steadily with increasing phenazine concentrations and exposure times, the transcriptional/translational (GFP production) activity demonstrated a “hormetic” (27) increase at toxin concentrations below 100 μM. The latter shape was matched by the Nrf2 activity curves in this subtoxic zone.

One feature that distinguishes between the cytotoxicity profiles of PYO and 1-HP is the cellular fate at high phenazine concentrations. All dose-response curves obtained for PYO show a complete shutdown of any cellular activities at >400 μM (Fig. 1), indicating total lethality. Indeed, in wells treated with 400 μM PYO, all cells appeared necrotic (Fig. S2). In contrast, BEAS-2B cells treated with 1-HP at 800 μM appeared rounded, but the cellular membranes in most cells appeared noncompromised (Fig. S2). As 1-HP doses increased up to 1 mM, the cellular viability leveled at 20 to 40% with respect to the untreated controls (Fig. 1), and lethality (defined as a >90% drop in cellular proliferation, or a viability value of <0.1) was not attained in the submillimolar range in BEAS-2B cells or either of the other two 1-HP-treated bronchial epithelial lines.

### Effects of polymyxins on bronchial epithelial cells.

Similar dose-response relationships were investigated for the polymyxin antibiotics with bronchial epithelial cells (Table 1 and Fig. 1). In contrast to the cytotoxic phenazines PYO and 1-HP, the polymyxins did not produce any significant activation of the Nrf2 transcription factor above the basal level in treated cells (Fig. 1). However, CMS produced hormesis-shaped dose-response curves in the 12-h and 24-h exposure experiments detecting GFP and luciferase activities, respectively. The values of the polymyxin concentrations producing a 50% inhibitory effect on mitochondrial metabolic and transcriptional/translational activities in the three bronchial epithelial lines are given in Table 1. Interestingly, in the set of tested polymyxins, CMS was the most toxic to BEAS-2B cells, while the reverse sensitivity to CMS was observed in HBE-1 and CFT-1 cells treated with the drugs for 24 h.

The observation that the cell “survival rate” in the colistimethate dose-response curves leveled at about 0.2 (Fig. 1), well into the millimolar range, suggests cellular arrest of BEAS-2B cells by CMS. A microscopic view of BEAS-2B cells treated with 1 mM CMS and 1 mM CST is given in Fig. S2 in the supplemental material. The CST-treated cells appear uniformly damaged, while the cellular response to CMS appears heterogeneous, with both intact-appearing and dead cells being observed in the wells after a 24-h exposure to colistimethate. This observation characterizes colistimethate, as well as 1-hydroxyphenazine, as cytotoxic, but not completely lethal, to bronchial epithelial cells under the conditions of our study.

### Interactions between polymyxins and phenazines.

In order to detect cytotoxic interactions (synergistic or antagonistic) in pairs of cytotoxic polymyxins and phenazines, we used the Chou-Talalay median-effect method (28), which is based on assumptions of mutual exclusivity of interacting drugs and a sigmoidal shape of the dose-response curves. The approach yields a combination index (CI) parameter that is calculated by using the equation CI = (*D*)_1_/(*D_x_*)_1_ + (*D*)_2_/(*D_x_*)_2_, where (*D_x_*)_1_ and (*D_x_*)_2_ are doses (concentrations) of individual drug 1 and drug 2, respectively, which individually produce the same effect as the combination of (*D*)_1_ plus (*D*)_2_. It follows from this equation, then, that for a pair of noninteracting drugs displaying the “additive effect,” the theoretical value for the CI is 1, while a CI of >1 and a CI of <1 would suggest, respectively, antagonistic and synergistic drug-drug interactions.

In this study, we have defined “effect” as a decrease in mitochondrial/metabolic activity or a decrease in transcriptional/translational activity, which was experimentally determined from fluorescence measurements in the resazurin and GFP reporter assays, such that effect = 1 − (RFU_treated_/RFU_control_), where RFU_treated_ is the relative fluorescence unit value for treated cells and RFU_control_ is the relative fluorescence unit value for control cells. In order to verify the applicability of the Chou-Talalay approach to our experimental setup, and due to a lack of information on the cytotoxicity mechanisms in the tested systems, we determined the experimental boundaries of the additivity effect by calculating CI values for a series of the “CMS-versus-CMS” and “PYO-versus-PYO” combinations. The results are listed in Table 2 and suggest that “additivity” between interacting drugs should be assigned for CI values falling roughly between 0.8 and 1.1.

**TABLE 2:** Combination indexes for phenazine-polymyxin combinations at the ED_50_ in three human bronchial epithelial cell lines^*a*^

Cell line	Assay	Mean CI ± SD for combination							
		CMS-CMS	PYO-PYO	CMS-PYO	CST-PYO	PMB-PYO	CMS–1-HP	CST–1-HP	PMB–1-HP
BEAS-2B	Resazurin	0.84 ± 0.16	0.99 ± 0.11	**0.27** ± 0.05	1.15 ± 0.04	1.20 ± 0.07	1.12 ± 0.21	0.86 ± 0.03	1.12 ± 0.02
	GFP	0.88 ± 0.10		**0.44** ± 0.06	1.51 ± 0.02	1.34 ± 0.08	1.05 ± 0.19	0.99 ± 0.26	0.95 ± 0.07
HBE-1	Resazurin	0.94 ± 0.05	1.10 ± 0.12	**0.59** ± 0.03	1.00 ± 0.06	1.15 ± 0.10	0.90 ± 0.06	1.07 ± 0.03	1.12 ± 0.19
CFT-1	Resazurin	1.00 ± 0.06	1.07 ± 0.13	**0.73** ± 0.02	1.20 ± 0.11	1.16 ± 0.11	**0.71** ± 0.04	0.90 ± 0.04	0.88 ± 0.02

a The theoretical CI value for additivity is 1, that for synergy is <1, and that for antagonism is >1. The CI values for “self-versus-self” interactions fall within the range of 0.84 to 1.1, thus defining the experimental additivity confidence interval. CI values for synergistic interactions are shown in boldface type for illustrative purposes. The errors are standard deviations for CI calculations based on at least three dose-response curves.

**FIG 2:**
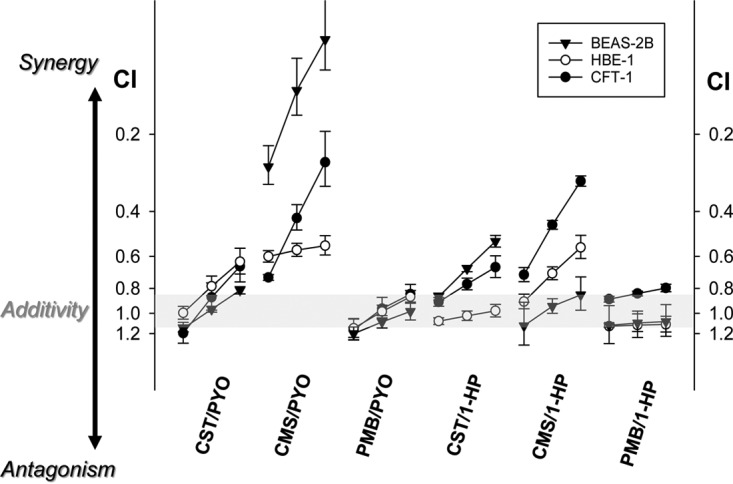
Combination indexes (CI) calculated for different levels of cytotoxicity effects. Each indicated drug combination was tested against BEAS-2B, HBE-1, and CFT-1 cells with the 24-h schedule. Cellular survival/proliferation was assayed with resazurin. The CI values were determined for 50%, 75%, and 90% cell inhibition effects; in each curve, these values are depicted by the left, central, and right points, respectively. The gray area indicates the additivity range, arbitrarily established from the “self-versus-self” combination data (CMS-CMS and PYO-PYO in Table 2). The error bars are SDs for at least 3 experiments.

We next determined CI values for all six possible combinations of cytotoxic phenazines and polymyxins, and the resulting values are listed in Table 2 and depicted in Fig. 2. It follows from these data that the combination of colistimethate and pyocyanin displays the strongest effect of synergistically enhanced cytotoxicity in all three cell lines, while polymyxin B lacks any significant interaction with either PYO or 1-HP. There is a uniform trend for CI values to decrease with an increasing cytotoxicity effect (Fig. 2), reflecting a steeper shape of the combination dose-response curves than of the single-agent curves. While BEAS-2B was the most sensitive cell line of the three for the CMS-PYO combination, the interaction between CMS and 1-HP was the strongest in the CFT-1 cell line.

**FIG 3:**
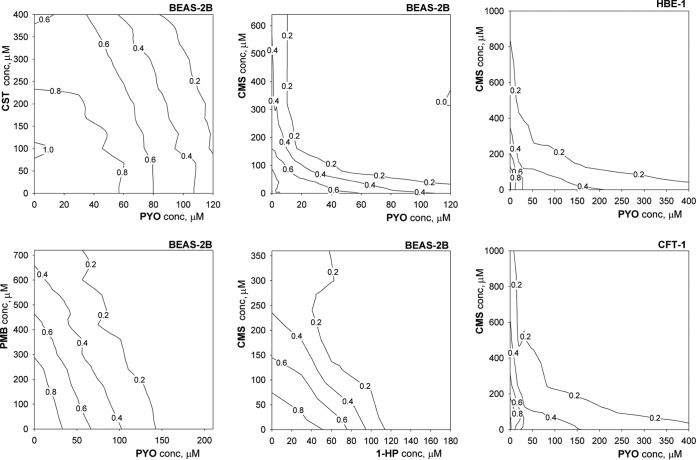
Isobolographic depiction of interactions in selected polymyxin-phenazine combinations following the 24-h drug exposure schedule. The isobole labels represent surviving fractions of cells treated with the combinations. A linear shape of the isoboles indicates additivity, while bending of the isoboles toward the origin point signifies a synergistic interaction; the opposite bend for the CST-PYO combination versus BEAS-2B cells suggests weak antagonism between the drugs.

An alternative approach to verify the conclusions of CI determinations is isobolographic analysis of the drug-drug combination response matrices (29). We performed a series of experiments for selected combinations and built the respective isobolograms (Fig. 3). Concavity of the isoboles signifies strong synergistic interactions, which are clearly apparent for the PYO-CMS combination treatments of all three cell lines at cell survival rates of 0.2 to 0.4. Straight isoboles are signatures of the additivity effect and occur with the diagram for the PMB-PYO (versus BEAS-2B) combination at all survival rates as well in the case of the CMS–1-HP (versus BEAS-2B) combination at most survival rates. Convexity of the isoboles indicates antagonistic drug-drug interactions and is observed at a high (0.8) cell survival rate for the CST-PYO (versus BEAS-2B) combination and, with lower confidence, at high survival rates for the CMS-PYO combination.

**FIG 4:**
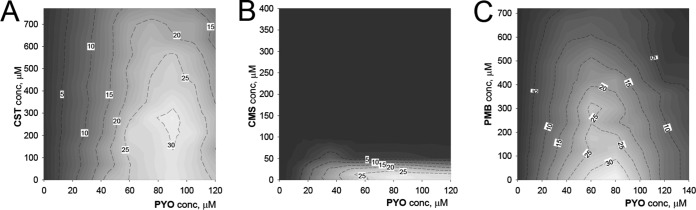
Activity of the Nrf2 transcription factor in BEAS-2B cells in response to 24-h exposures to combinations of CST-PYO (A), CMS-PYO (B), and PMB-PYO (C). The contour labels and the background color denote fold increases in luminescence relative to the values for untreated control wells.

We also employed the drug-drug combination response matrices for tracking activation patterns of the transcriptional factor Nrf2. As illustrated in Fig. 4, colistimethate at subtoxic concentrations (100 μM) suppressed Nrf2 activity (visualized in Fig. 4B by light/dark color contrast) in BEAS-2B cells treated with PYO. In contrast, neither CST nor PMB significantly interrupted Nrf2 activation until it reached the 50% effective dose (ED_50_).

To assess potential interactions between polymyxins and low-toxicity phenazine-1-carboxylic acid and phenazine-1-carboxamide, we applied the effect-based Bliss independence approach (30). This experiment was designed as a polymyxin cytotoxicity potentiation study; i.e., the dose-response curves were built for polymyxins in the presence of fixed concentrations of PCA and PCN, arbitrarily chosen to be 120 μM. The normalized ED_50_ and CI values are given in Table 3. According to the data, both PCA and PCN at 120 μM potentiate the cytotoxicity of colistimethate while mildly counteracting polymyxin B effects.

**TABLE 3:** Normalized ED_50_s calculated from mitochondrial metabolic (resazurin) and transcriptional/translational (GFP) activities in BEAS-2B cells treated with polymyxins in the presence of low-toxicity phenazines

Parameter	Value for assay^*c*^					
	Resazurin			GFP		
	No phenazine	120 μM PCA	120 μM PCN	No phenazine	120 μM PCA	120 μM PCN
Mean survival base ± SD^*a*^	1 ± 0.04	0.95 ± 0.04	0.84 ± 0.07	1 ± 0.04	0.98 ± 0.04	0.98 ± 0.03
Mean ED_50_ (μM) ± SD (CI) for antibiotic^*b*^						
Colistimethate	178 ± 15	**112± 31**(0.65)	**147± 14**(0.77)	253 ± 24	**134± 18**(0.37)	**169± 43**(0.44)
Colistin	468 ± 23	488 ± 20 (0.93)	**404± 35**(0.83)	696 ± 36	711 ± 33 (1.01)	691 ± 27 (0.95)
Polymyxin B	545 ± 6	**659± 26**(1.22)	**629± 32**(1.12)	649 ± 22	**740± 23**(1.24)	697 ± 56 (1.09)

a Fraction of surviving cells in untreated wells (controls) or wells treated with 120 μM phenazines only.

b Combination index values for the Bliss independence model at the ED_50_ are given in parentheses. A CI of <1 is indicative of potentiation, and a CI of >1 implies moderation of cytotoxicity.

c The errors are standard deviations from at least 3 determinations; the combination ED_50_ values in boldface type are significantly different from the respective ED_50_ values obtained for polymyxin-only treatments.

### Chelators protect cells from pyocyanin cytotoxicity.

**FIG 5:**
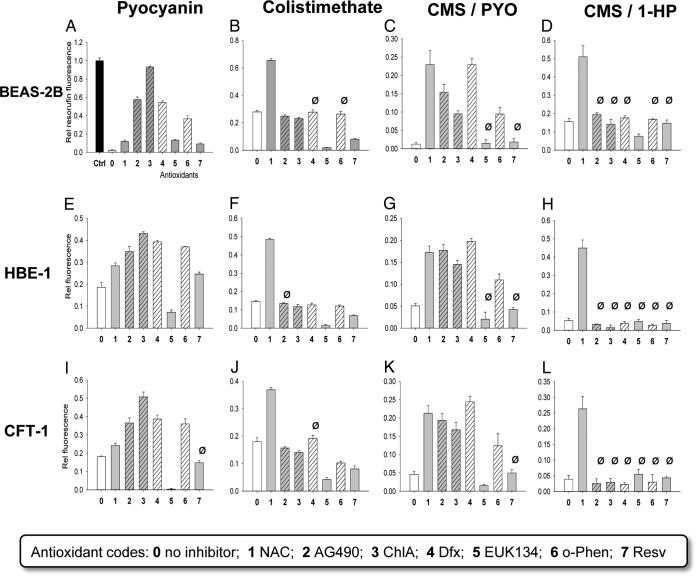
Effects of antioxidants on cytotoxicity of pyocyanin (PYO), colistimethate (CMS), and the CMS-PYO and CMS–1-hydroxyphenazine (1-HP) combinations in three human bronchial epithelial cell lines, as measured after a 24-h exposure in the resazurin assay. Gray bars correspond to antioxidants that are capable of reducing reactive oxidants, and slanted patterned bars correspond to strong iron chelators. Specific concentrations of the cytotoxic agents are 400 μM PYO (A), 600 μM PYO (E and I), 600 μM CMS (B), 1 mM CMS (F and J), 300 μM CMS–200 μM PYO (C), 500 μM CMS–200 μM PYO (G and K), and 300 μM CMS–100 μM 1-HP (D, H, and L). Specific concentrations of antioxidants are 10 mM *N*-acetyl-cysteine (NAC), 50 μM tyrphostin AG490 (AG490), 100 μM chlorogenic acid (ChlA), 200 μM deferasirox (Dfx), 100 μM EUK134 (cell-permeable catalase/SOD mimic), 50 μM *o*-phenanthroline (*o*-Phen), and 100 μM resveratrol (Resv). The error bars are SDs for 3 experiments. Statistical significance between the “no-inhibitor” and antioxidant effects was probed by analysis of variance; for clarity, Ø denotes statistical nonsignificance (*P* ≥ 0.05).

Having established (Fig. 1 and 4) that PYO and 1-HP activate the transcription factor Nrf2, which is responsive to intracellular oxidative stress (31), we next asked whether their toxicity in bronchial epithelial cells could be modulated by antioxidants. The bacterial phenazine PYO can cause a surge in the levels of intracellular reactive oxygen species (ROS) and oxidative stress due to its redox cycling ability (21), while iron may be required as a mediator of such activity (32). Potential inhibitors of phenazine-induced oxidative stress would therefore possess one or more of the following chemical properties: (i) quenching ROS, (ii) binding a metal redox catalyst, and/or (iii) reducing oxidized intracellular proteins and lipids back to the original state. Acknowledging this, we assembled a short panel of small-molecule antioxidants (Fig. 5), representing the above-mentioned modes of antioxidant action. The panel was evaluated with three bronchial epithelial cell lines for protective effects against lethal doses (survival fraction of <0.2) of PYO, CMS, and the CMS-PYO and the CMS–1-HP combinations. As shown in Fig. 5, only 10 mM *N*-acetyl-cysteine (NAC) offered significant protection to CMS-treated cell lines. Antioxidants capable of binding iron, i.e., the nonreducing chelators deferasirox (Dfx) and *o*-phenanthroline (*o*-Phen), as well as the reducing catechol derivatives chlorogenic acid (ChlA) and tyrphostin A490, were better at protecting PYO-treated cells. Both NAC and the iron-binding antioxidants significantly improved the survival of cells treated with the CMS-PYO combination. In contrast, the nonchelating phenolic antioxidant resveratrol (Resv) and the cell-permeable catalase/superoxide dismutase (SOD) mimic EUK134 were ineffective in all experiments. *N*-acetyl-cysteine at 10 mM was the only antioxidant able to protect all three cell lines from the cytotoxic CMS–1-HP combination.

## DISCUSSION

The identification of potential interactions between toxic agents in living organisms, from humans to crops to pathogens, is one of the important tasks of experimental pharmacology and toxicology (33, 34). Accumulated knowledge in this area suggests that such interactions, synergistic or antagonistic, are likely to occur between agents acting along similar signaling, metabolic, or physiological pathways (35, 36). Inhalations of colistimethate, both powdered and solubilized, cause a number of adverse effects in the airways of a significant proportion of CF patients chronically infected with P. aeruginosa (12, 13), with occasional reports of hypersensitivity and severe bronchospasms (9, 37). Toxic P. aeruginosa metabolites, such as PYO and 1-HP, were reported to induce pulmonary pathophysiology features characteristic of CF as well (24, 38). This background provided a rationale for the idea that polymyxins can interact with P. aeruginosa phenazines against pulmonary epithelial cells, and the hypothesis was put to the test in the present study.

This study established that all three polymyxin antibiotics, namely, colistin, colistimethate, and polymyxin B, significantly inhibit mitochondrial, total transcriptional/translational, and Nrf2-mediated stress response activities *in vitro*, at concentrations that can be clinically achievable in the airways. Thus, maximal concentrations of colistimethate delivered by the intrapulmonary route in human and experimental animal lung fluids were reported to be ∼3 g/liter (about 2 mM [14]) and ∼10 g/liter (in rat [39]), respectively; the corresponding maximal levels of formed CST in these studies were 1 g/liter and 2 g/liter, respectively. Two out of four P. aeruginosa phenazines, PYO and 1-HP, were cytotoxic at high but physiologically relevant, at least for PYO, concentrations. When the polymyxin-phenazine pairs were tested against three bronchial epithelial cell lines (18 combinations in total), most of the combinations showed a lack of significant cytotoxic interactions, but the CMS-PYO pair showed a surprisingly strong effect of synergistically enhanced toxicity. Given that CMS, rather than colistin or polymyxin B, is the pharmaceutical form of polymyxin delivered by inhalation to P. aeruginosa-infected airways, our findings appear to be important.

Using the ED_50_ after 24 h of exposure as an endpoint, we first obtained evidence that all polymyxins are cytotoxic to bronchial epithelial cells *in vitro* at submillimolar concentrations, which are reasonably achievable in airways of patients taking the drugs by inhalation. A number of clinical and laboratory pharmacokinetic studies (14, 39, 40) demonstrated that high (≥1 mM) concentrations of aerosol-dosed CMS, along with formed colistin, can remain in airway fluids for hours. For example, in one clinical study (14), concentrations of both CMS and formed colistin in the epithelial lining fluid (ELF) of patients who received a single dose of 10 mM CMS via aerosol were relatively stable for about 6 h and amounted to up to 1.4 and 1.1 g/liter (∼1 mM), respectively. On the other hand, the higher cytotoxicity of CMS than of CST and PMB in BEAS-2B cells is surprising, in view of data obtained in another *in vitro* study (8) as well as a number of toxicological studies that cite CMS as being the safest of the three drugs (2). One possible explanation for this discrepancy may be that CMS cytotoxicity is due, at least in part, to a product(s) of its hydrolysis other than CST. One molecule of CMS contains five sulfomethylamino groups, which are known to undergo hydrolysis with the release of formaldehyde (41). Formaldehyde is highly cytotoxic but is readily detoxified by serum proteins when in circulation. Our *in vitro* experiments were performed with serum-free medium, and CMS cytotoxicity could be inhibited only by *N*-acetyl-cysteine, which is an efficient formaldehyde-trapping reagent (42). Other proposed products of CMS hydrolysis are tetra-, tri-, bi-, and monosulfomethylated colistin species, which may form at concentrations exceeding those of formed CST (43). However, no toxicological data are available for these intermediates so far.

The physiological relevance of our *in vitro* model for the evaluation of phenazine cytotoxicity has been verified as well. The ED_50_s for PYO and 1-HP varied within the range of 80 to 200 μM, which are within the range of concentrations physiologically achieved in the airways (i.e., as measured in sputa from cystic fibrosis patients, up to 27 μg PYO/g sputa, equaling 130 μM PYO) (15). Two other phenazine metabolites of P. aeruginosa, PCA and PCN, were not cytotoxic in our experiments. Nevertheless, these molecules are capable of inducing, along with PYO, proinflammatory responses in pulmonary cells (22, 44) and thus could contribute to the pathophysiology of P. aeruginosa infections in airways.

One interesting observation of this study is the cytotoxicity profiles of CMS and 1-HP at higher concentrations of the agents, which suggest cellular senescence rather than total kill. When combined, however, CMS and 1-HP produced moderately synergistic cytotoxicity effects and actual cell death in HBE-1 and CFT-1 cells.

The most interesting combination is the colistimethate-pyocyanin pair, since it showed the strongest synergistic interaction against all three airway cell lines. This observation is rather unexpected, as the main suggested mechanism of pyocyanin cytotoxicity in epithelial cells is oxidative stress (21, 32). In contrast, the cellular stress induced by CMS and other polymyxins seemingly took a different pathway, as evidenced by the lack of Nrf2 activation (Fig. 1). One clue about a possible CMS-PYO interaction mechanism is provided by the behavior of Nrf2 in cells treated with polymyxin-PYO combinations (Fig. 4). The unambiguous suppression of PYO-induced Nrf2 activation by CMS, but not CST or PMB, in BEAS-2B cells may be mechanistically related to the ability of CMS to sensitize cells to PYO cytotoxicity. For example, profiling of the CMS-PYO dose-response matrix (Fig. 3) along a constant subtoxic CMS concentration of 50 μM yields a dose-response curve for PYO with a calculated ED_50_ of 16 μM. This value is >5-fold lower than the ED_50_ of 87 μM produced by PYO alone (Table 1). It is known that mammalian cells are able to naturally counteract PYO effects through the maintenance of redox homeostasis (45) or the oxidative degradation of this phenazine (46). The observation of a hormetic shape of the dose-response curves for transcriptional/translational activity (Fig. 1), that is, the “paradoxical” increase of activity at low concentrations of PYO and 1-HP, hints at processes of cellular adaptation to these toxins. Hormesis is considered a hallmark of cellular adaptation through receptor or cell signaling pathways (27). Since intracellular antioxidant defense is regulated mainly by KEAP1/Nrf2 transcriptional activity, inhibition of this pathway could weaken the cellular defense against PYO. On the other hand, the nontoxic phenazines PCA and PCN appeared to potentiate the cytotoxicity of CMS as well. Detailed mechanistic considerations of these interactions would require special investigation beyond the goals of this initial study.

Some additional mechanistic insights can be drawn from the inhibitor experiment. While many research groups over the years have described PYO-induced intracellular oxidative stress (20, 47, 48), we recently identified iron as an essential mediator in the pyocyanin-catalyzed autoxidation of intracellular thiols in renal tubular epithelial cells (32). Indeed, strong iron chelators appeared to be the single most potent inhibitors of pyocyanin cytotoxicity in kidney epithelial (32) and lung epithelial (this study) cells. CMS cytotoxicity was effectively inhibited by *N*-acetyl-cysteine, a protector of cellular thiol homeostasis. It may be hypothesized, then, that a mechanism of the synergistically enhanced cytotoxicity of the CMS-PYO combination is related to the ability of both CMS and PYO to target the intracellular thiol pool albeit by different chemistries: the reactive oxidation (catalyzed by PYO) and, possibly, the reactive carbonyl species (released by CMS).

Our study has several limitations, as would be expected for any *in vitro* model. The first limitation is a lack of information on the fate of CMS during the experiments. CMS is a product of colistin sulfomethylation at its five amino groups. Although CMS hydrolysis can proceed spontaneously in pure water, it accelerates dramatically in the presence of buffers and especially in biological milieu, such as blood serum. Besides partially hydrolyzed CMS and colistin, no other products of the hydrolysis reaction or enzymatic/nonenzymatic catalysts have been identified, to the best of our knowledge. Thus, the CMS hydrolysis mechanism is not known, and its rates in different media or intracellularly cannot be controlled or predicted. Indeed, the variability of hydrolysis rates of CMS delivered by the intrapulmonary route in lungs of patients or experimental animals spans over several orders of magnitude, both within and between studies (14, 39, 40). The second limitation of our study is the significant difference in physicochemical characteristics between the experimental cell culture medium used *in vitro* and the airway medium in healthy or infected lungs, which is also characterized by heterogeneity (mucus-periciliary fluid) and a great variability in chemical composition, viscosity, or the population of bacterial and immune cells (49). Next, for any intravenously (i.v.) delivered polymyxin drug, its reported concentration in airway fluids had never exceeded the 30 μM (maximum concentration of drug in lung epithelial lining fluid [*C*_max_] of ∼30 mg of formed CST per liter, for example [14]) barrier, at which point the polymyxin drug cytotoxicities were not significant in our experiments. In plasma, concentrations exceeding 50 mg/liter have not been reported for any polymyxin drug either, regardless of the drug delivery route. In addition, bacterial phenazines in the circulation have not yet been determined. Therefore, the clinical relevance of our observations is restricted to the airway fluid environment in a host with a chronic, PYO-positive P. aeruginosa infection that is treated with CMS by pulmonary delivery.

The observations of enhanced cytotoxicity of CMS and P. aeruginosa phenazines when these compounds occur together, as would be anticipated in cystic fibrosis airways, deserve further investigations. Among these would certainly be establishing whether the phenomenon is of any clinical significance. In animal models and *in vitro*, elucidation of the synergistic cytotoxic interactions in physiological settings and in different cell types, clarification of the mechanism(s), and a search for effective inhibitors would be of importance as well.

In conclusion, both polymyxin antibiotics and bacterial phenazines cause cytotoxic stress in human bronchial epithelial cells at physiologically achievable concentrations *in vitro*. Colistimethate can interact with pyocyanin, the most common P. aeruginosa VF phenazine, by a synergistic increase in toxicity to cells. PYO cytotoxicity alone or in combination with CMS can be attenuated, to some degree, by iron chelators, while *N*-acetyl-cysteine could diminish the cytotoxicity of CMS in the presence of phenazines. These results provide a rationale for further evaluation of potentially hazardous colistimethate interactions in relevant laboratory and clinical models of P. aeruginosa infections.

## MATERIALS AND METHODS

### Reagents.

Non-pharmaceutical-grade antibiotics included colistin sulfate (∼20,000 U/mg; Chem-Impex), colistin sodium methanesulfonate (CMS) (from Bacillus colistinus; Fluka), and polymyxin B sulfate (∼8,000 U/mg; Research Products International). Stock solutions of CMS were kept frozen until use. The reagent-grade phenazines 1-hydroxyphenazine (1-HP), phenazine-1-carboxylic acid (PCA), and phenazine-1-carboxamide (PCN) were obtained from ArkPharm and were used without further purification. Crystalline pyocyanin was purchased from Cayman or prepared by photolysis of a 2 mM solution of phenazine methosulfate in 20 mM HEPES buffer (pH 7.4), as reported previously (32). In-house nanopure water was generated by double distillation and used in all experiments. Antioxidants employed in this study included *N*-acetyl-l-cysteine (NAC), tyrphostin AG490, chlorogenic acid (ChlA), deferasirox (Dfx), the cell-permeable catalase/SOD mimic EUK134, *o*-phenanthroline (*o*-Phen), and resveratrol (Resv).

### Cell culture.

The normal human bronchial epithelial cell line BEAS-2B (50) was purchased from the American Type Culture Collection. The original BEAS-2B line and the reporter line BEAS-2B.R05Z were routinely cultured in a 1:1 Dulbecco's modified Eagle's medium (DMEM)–Ham F-12 medium (Sigma) mixture supplemented with 2% newborn calf serum (NCS; HyClone); 2 mg/liter insulin, 2 mg/liter transferrin, and 2 μg/liter selenite (2-ITS) (all from Sigma); and a 1% (vol/vol) penicillin-streptomycin cocktail (HyClone). The immortalized human cystic fibrosis bronchial epithelial line CFT-1 and a matching normal cell line, HBE-1 (51), were obtained from Kerafast. CFT-1 and HBE-1 cells were cultured in bronchial epithelial cell growth medium (Lonza). The cells were subcultured at a 1:5 ratio upon reaching near confluence. The standard culturing conditions for all cells were 37°C with 5% CO_2_ and 100% humidity.

### Plasmid constructs.

**FIG 6:**
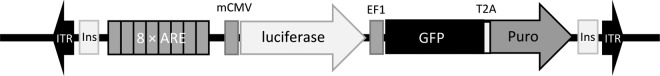
The transcriptional activity reporter construct. A block of eight antioxidant/electrophile response elements, the sites for binding the cellular stress master regulator Nrf2, is followed by a minimal cytomegalovirus (mCMV) promoter that is followed by a Brazilian click beetle green luciferase gene. In addition, the EF-1 promoter provides continuous transcription of destabilized green fluorescent protein and puromycin resistance genes. A pair of flanking insulators (Ins) protects the construct from epigenetic silencing (25), while the *piggyBac* transposon inverted terminal repeats (ITR) provide a clean insertion of the reporter into transcriptionally active regions of cellular chromatin.

The Super *piggyBac* transposase expression vector was purchased from System Biosciences. Vector pCBG99 was obtained from Promega. Reporter vector pTR05Z was prepared by inserting the Brazilian click beetle luciferase gene from pCBG99 into an insulated *piggyBac* transposon construct containing an 8× antioxidant/electrophile transcriptional response element (ARE) upstream and a copepod green fluorescent protein (GFP) gene downstream of the insert, as shown in Fig. 6. The resulting plasmid, pTR05Z (see Fig. S3 in the supplemental material), was verified by DNA sequence analysis.

### Transfections.

In order to generate the stable reporter line BEAS-2B.R05Z, the original BEAS-2B cells were seeded into wells of a 96-well plate at 2 × 10^4^ cells per well in antibiotic-free DMEM–F-12 medium supplemented with 5% NCS and left to adhere for 6 h. The cells were then treated with a mixture of 100 ng pTR05Z reporter plasmid and 33 ng the Super *piggyBac* transposase plasmid complexed with TransIT X2 transfection reagent (Mirus) at a ratio of 1:2 (micrograms of DNA per microliter). After 16 h, regular media were added, and the cells were left to proliferate for the next 48 h. The transfected cells were then treated with the selecting antibiotic (5 μg/ml puromycin) for another week, and the surviving cells were expanded for cryopreservation and activity validation.

### Cell treatment schedule.

Typically, BEAS-2B cells or their reporters were plated into 96-well plates (BioLite; Fisher) at 1 × 10^4^ cells/well in 100 μl of the culture medium. After 48 h, the proliferation medium was replaced with phenol red-free serum-free (SF) medium (Corning) supplemented with the 2-ITS mixture and penicillin-streptomycin (test medium). Corning SF medium is a 1:1 mixture of DMEM–F-12 medium containing smaller proportions of RPMI 1640, McCoy's 5A medium, and 1 g/liter bovine serum albumin. The cells were cultured for the next 24 h, after which the medium was replaced with fresh test medium containing cytotoxic agents or carriers and the cells were incubated for 24 h or as indicated. In some cases, the cells were treated with single agents for 12 h and then allowed to recover in drug-free test media for the next 12 h, before being further processed for cell proliferation, as described below.

HBE-1 and CFT-1 cells were plated into 96-well plates at 1 × 10^4^ cells/well in 100 μl of adaptation medium, which consisted of a 3:1 mixture of BEGM and Corning serum-free test medium. After 48 h, this medium was replaced with a 1:4 mixture of BEGM-Corning SF test medium, and the cells were cultured for the next 24 h, before being treated with the agents described above.

Cell survival and proliferation were evaluated by either (i) mitochondrial metabolic activity (resazurin reduction) or (ii) transcriptional/translational activity (GFP expression). To measure mitochondrial/metabolic activity, adherent treated cells were washed and incubated at 37°C for 30 min with 70 μl of the test medium containing 10 mg/liter resazurin. The fluorescence of 60-μl aliquots, along with blanks, was recorded at an excitation (Ex) wavelength of 540 nm and an emission (Em) wavelength of 590 nm. The fluorescence in untreated wells was assigned a 100% survival rate and used to normalize the proliferation rates in treated wells. Cell proliferation rates were also evaluated by GFP fluorescence, as a part of the reporter activity assay (see below).

### Nrf2 reporter activity assay.

In a typical experiment, immediately after treatments or following the 30-min resazurin run, the reporter cells were washed and lysed in 70 μl of luciferase reporter lysing buffer (Promega). The lysate fluorescence was measured with a setup consisting of an Ex wavelength of 482 nm (slit width, 9 nm)/Em wavelength of 512 nm (slit width, 17 nm). This was followed by the addition of the luciferase substrate (Promega) and kinetic luminescence readings in the wells, which were done in 2-min intervals for 8 min total. All the measurements were done by using a Synergy MX (BioTek) multimode plate reader. The GFP fluorescence values were used for both the evaluation of relative cell transcriptional/translational activity (proliferation) and normalization of the Nrf2 reporter luciferase activities in the respective wells (25).

### Analysis of dose-response curves.

Analysis of dose-response curves was performed by using CalcuSyn software (Biosoft), based on the Chou-Talalay median-effect method (28). Dose-response curves lacking the sigmoid shape at low agent doses due to the hormetic effect or high agent doses due to cellular senescence were truncated: only data points from the steep regions around the ED_50_ were used for calculations. For interacting agents, as described in more detail in Results, the calculated combination index (CI) implies synergism and antagonism of the interacting drugs at CI values of <1 and >1, respectively.

The combination effects were visualized by isobolographic analysis (29) of dose-response surfaces for two drug combinations, whereby the theoretical additivity isobole yields a straight line and synergistic or antagonistic interactions produce isoboles that are curved, correspondingly, inwards or outwards with respect to the origin.

The Bliss independence-effect-based model (30) was also employed to evaluate the potentiation of a cytotoxic drug by a nontoxic or low-toxicity dose of the second agent. In this model, the combination index is calculated based on the assumption that the effect of one drug (*E*_1_) is independent of the effect of the second drug (*E*_2_), as they combine to produce a common result (*E*_12_), so that CI = (*E*_1_ + *E*_2_ − *E*_1_*E*_2_)/*E*_12_.

### Plotting and statistical analysis.

Statistical tests and plots were done by using SigmaPlot (version 11.0) and CalcuSyn.

## Supplementary Material

Supplemental file 1
