# Supplementary material for: Interaction of Bacterial Phenazines with Colistimethate in Bronchial Epithelial Cells
Source: Antimicrob Agents Chemother. 2018 Jul 27;62(8):e02349-17. doi: 10.1128/AAC.02349-17 (PMC6105780; doi:10.1128/AAC.02349-17)
Supplement: Supplemental file 1 [file zac008187311s1.pdf]

Interaction of Bacterial Phenazines with Colistimethate in Bronchial Epithelial Cells

By Valeri V. Mossine,[a,b]# Deborah L. Chance,[c,d] James K. Waters,[b] Thomas P. Mawhinney[a,b,d]

Departments of [a]Biochemistry, [c]Molecular Microbiology and Immunology, and [d]Child Health, University of Missouri, Columbia, Missouri, USA

[b]Experiment Station Chemical Laboratories, University of Missouri, Columbia, Missouri, U.S.A.

#Address correspondence to Dr. Valeri V. Mossine, MossineV@missouri.edu

Supplementary Figures S1-S3

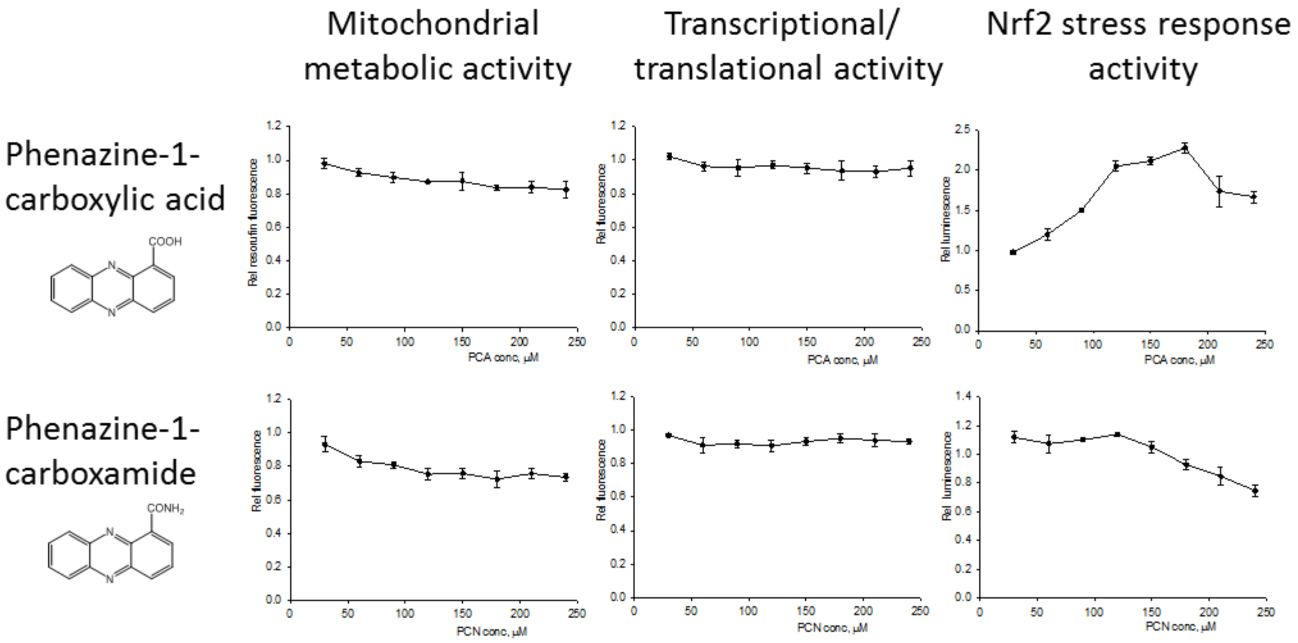

Figure S1. The cytotoxicity of *P. aeruginosa* phenazines in BEAS-2B cells exposed to the agents for 24 h. The mitochondrial metabolic activity was evaluated fluorimetrically by measuring rates of resazurin reduction. The transcriptional/ translational activity was assessed by determination of destabilized GFP in cell lysates. The relative activity of the stress responsive transcriptional factor Nrf2 was calculated by normalizing the luminescence readings with the GFP fluorescence values from the same wells. The error bars are SDs for at least 3 biological samples.

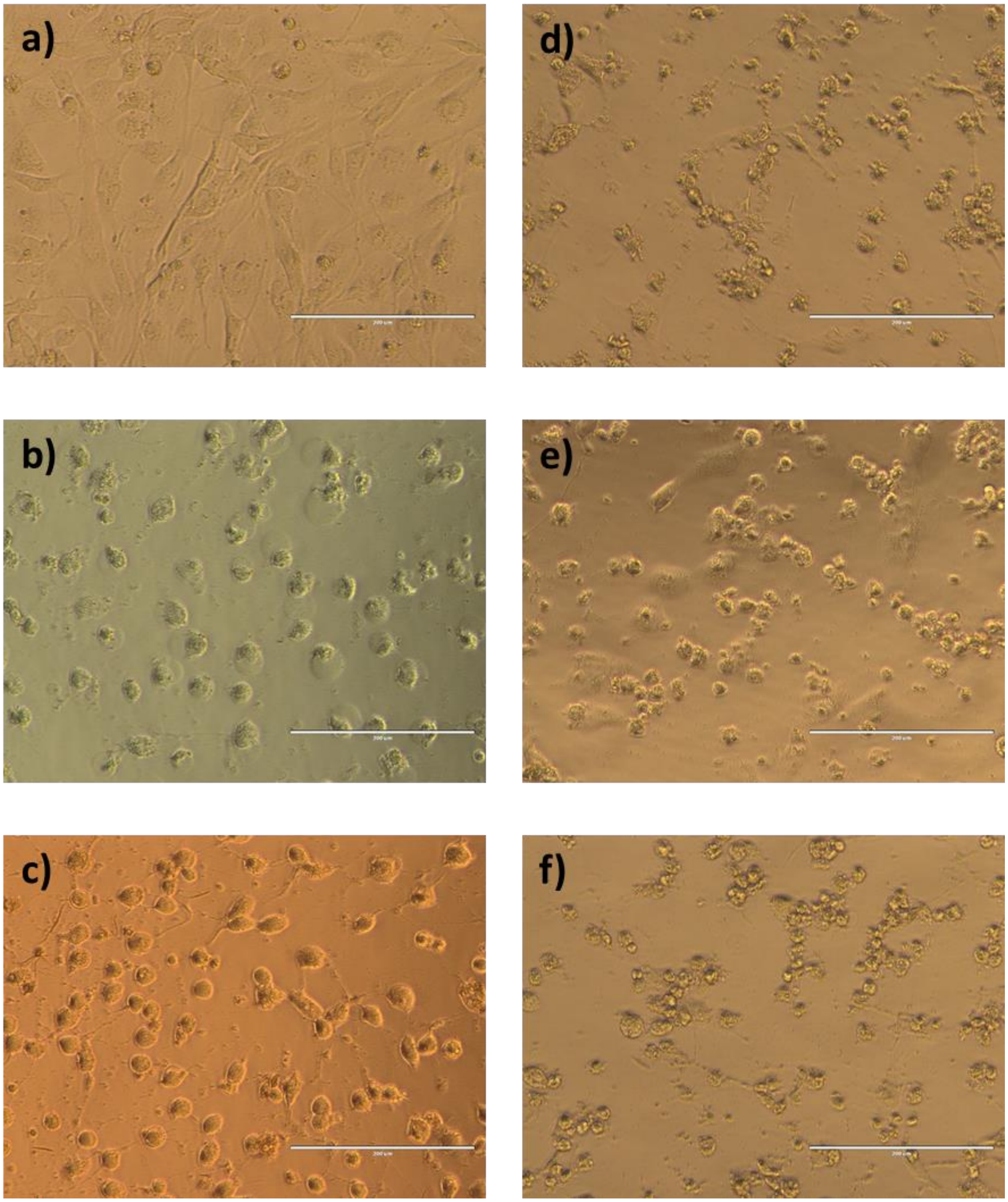

Figure S2. BEAS-2B cells were treated for 24-hour with: **a)** no agents; **b)** 400 µM PYO; **c)** 800 µM 1-HP; **d)** 1 mM CST; **e)** 1 mM CMS; **f)** 200 µM 1-HP/ 300 µM CMS. The scale bar: 200 µm.

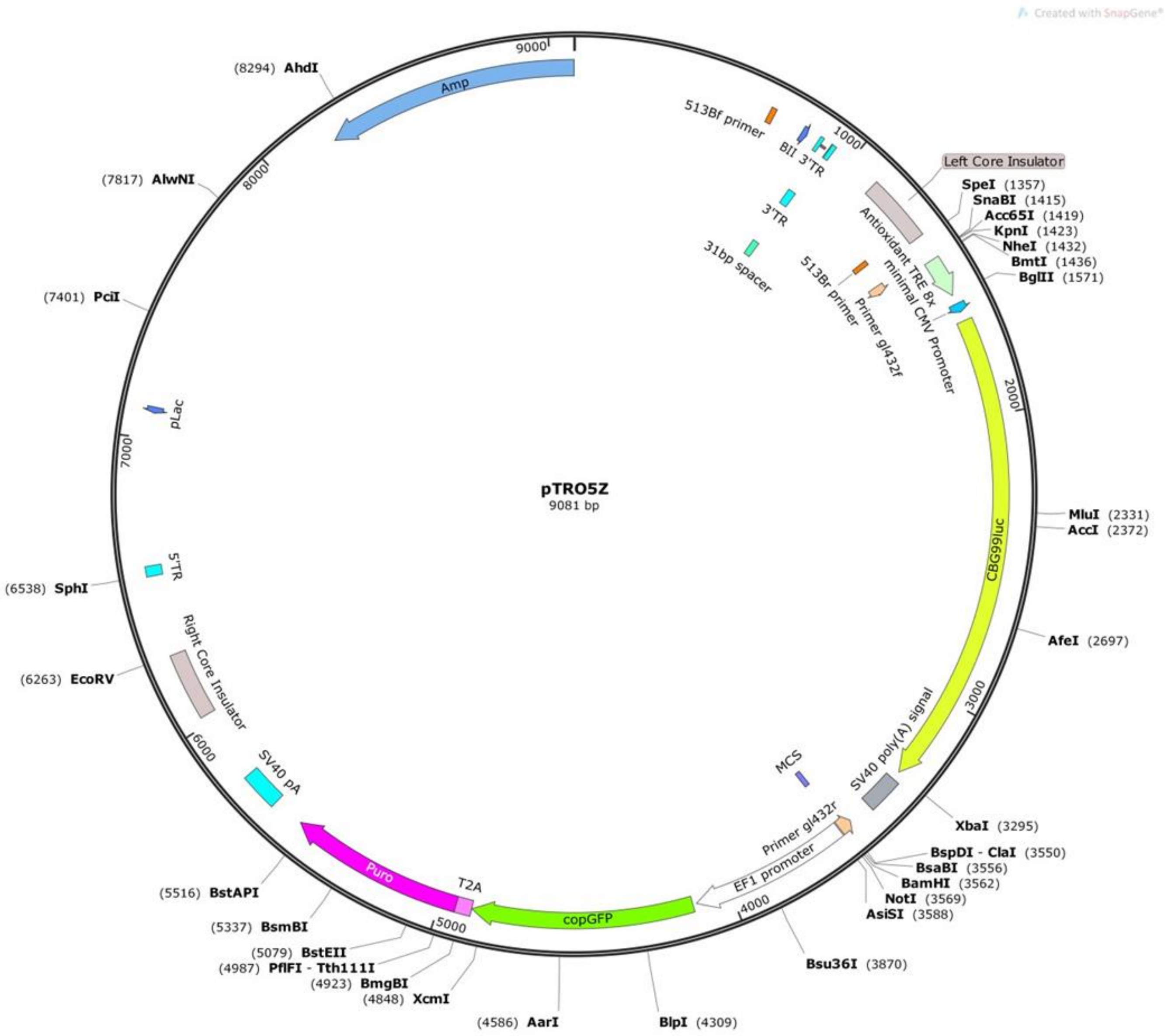

Figure S3. pTR05Z vector construct
